# GaitSpoofNet: advanced spatio-temporal architectures for vision-based presentation attack detection

**DOI:** 10.3389/frai.2026.1821341

**Published:** 2026-06-30

**Authors:** Islam Mohamed, Ahmad Salah, Essam Debie, Marwa Abdellah, Amr Abdellatif

**Affiliations:** 1 Department of Computer Science, Faculty of Computers and Informatics, Zagazig University, Zagazig, Egypt; 2 Department of Computing and Information Sciences, College of Computing and Information Sciences, University of Technology and Applied Sciences, Ibri, Al Dhahirah, Oman; 3 School of Information Technology and Systems, University of Canberra, Canberra, ACT, Australia

**Keywords:** biometric security, comparative study, deep learning, gait anti-spoofing, gait spoofing detection, GRU, LSTM, Mamba

## Abstract

**Introduction:**

Gait recognition offers a promising non-intrusive biometric modality, but its widespread adoption is critically hindered by its vulnerability to spoofing attacks, also known as Presentation Attacks (PAs). Developing effective gait anti-spoofing, or Presentation Attack Detection (PAD), mechanisms is therefore paramount for the security and reliability of gait-based authentication systems. While anti-spoofing research in gait has been addressed across both sensor-based (accelerometer/gyroscope) and vision-based (silhouette/image) modalities, this work specifically focuses on vision-based, image-level gait PAD, a domain that critically lacks dedicated deep temporal models and standardized benchmarks. Gait spoofing is defined as deliberate manipulation of a subject's external appearance to deceive the authentication system.

**Methods:**

Unlike prior work, we evaluate models under two practical scenarios: a public-access environment (random splitting) and a restricted-access scenario (LNSOCV). We present a comprehensive comparative study of advanced spatio-temporal architectures for gait anti-spoofing. By repurposing the CASIA-B dataset, we establish a standardized vision-based PAD baseline. We systematically evaluate models incorporating the official Mamba Selective State Space Model (mamba-ssm), a custom Inspired Mamba architecture, Gated Recurrent Units (GRU), and Long Short-Term Memory (LSTM) networks, all leveraging a robust CNN backbone.

**Results:**

Our extensive experiments demonstrate that all investigated advanced temporal models significantly improve gait spoofing detection over baseline methods. In open-access environments, the GRU-based model proved to be the most effective for anti-spoofing, reaching a state-of-the-art final validation accuracy of 0.9840 and an ROC-AUC of 0.9983.

**Discussion:**

Under restricted-access conditions, the LSTM-based model demonstrated the strongest overall performance.

## 1 Introduction

Gait, the manner of human walking, has emerged as a distinctive biometric trait. However, its practical application in security systems is critically dependent on addressing its vulnerability to spoofing. Its unobtrusive nature makes gait attractive for surveillance (Liu et al., 2025) and continuous authentication (Giorgi et al., 2021), yet spoofing attacks–where adversaries present fake gait patterns (e.g., mimicking, replaying videos)—can severely compromise system integrity. Robust gait anti-spoofing is thus a fundamental requirement. Spoofing attacks in computer vision can be categorized into several types depending on the targeted biometric system.

Most published work on gait anti-spoofing relies on inertial sensors—accelerometers or gyroscopes embedded in smartphones or dedicated wrist bands (Kumar et al., 2023; Mekruksavanich and Jitpattanakul, 2024; Randombage and Jayawardene, 2024; Salvador-Ortega et al., 2023; Chen et al., 2024; He et al., 2026). These sensors capture three-axis acceleration directly, giving them a physical grounding in true biomechanics that is difficult to spoof without replicating the walker's exact force profile. Their limitation is deployment: every subject must carry a sensor, ruling out unobtrusive or retrospective monitoring. Our work takes a different starting point—standard CCTV silhouette sequences—because that infrastructure already exists in the settings we care about. The cost is that we must distinguish appearance-based attacks purely from image geometry, with no force or acceleration signal available. Spoofing attacks have been studied across face (Maatta et al., 2011; Zheng et al., 2024; Lin et al., 2025), fingerprint (Wone et al., 2025; Cheniti et al., 2025; Lokhande et al., 2024), iris (Narkar and David-John, 2024; Zhang T. et al., 2024; Gupta et al., 2014), and gait (Boulgouris et al., 2006) biometrics. This study focuses specifically on gait-based spoofing attacks. Various types of datasets are used for gait recognition, such as RGB images (Chattopadhyay et al., 2014), depth images (Sivapalan et al., 2011), infrared images (Liu et al., 2024), silhouettes (Peng et al., 2023), and sensor data (Sezavar et al., 2024). Most gait recognition models utilize silhouette data as input. Accordingly, widely used silhouette datasets such as CASIA-B are repurposed in this study to address the gait spoofing problem. In this study, we define gait spoofing as the deliberate alteration of an individual's external appearance—specifically through clothing changes or the carrying of objects—with the intent to deceive the gait-based authentication system, a form of presentation attack as explored in Hadid et al. (2012), Hadid et al. (2013), Bustard et al. (2014), Hadid et al. (2015), and Masood and Farooq (2017). Gait recognition is particularly suited for low-resolution and long-distance identification where face, fingerprint, or iris recognition are infeasible. Yet few studies discuss gait spoofing or its security implications. Additionally, while sensor-based countermeasures exist, there is no publicly available deep learning model that practically addresses this problem for vision based, silhouette-reliant systems.

Detecting gait Presentation Attacks (PAs) is challenging due to subtle spoofing cues and diverse attack methods. Effective PAD systems must distinguish genuine motion from manipulated presentations through complex spatio-temporal analysis. While deep learning has been applied to gait recognition, there are currently no public, dedicated deep learning implementations specifically for silhouette-based (vision-based) gait spoofing. Simpler CNN-RNN approaches may lack capacity for long-range temporal dynamics, leading to insufficient generalization against novel attacks (Asharf et al., 2020). Well-established frameworks and benchmarks for gait spoofing detection remain scarce, with current literature focused on identification rather than PAD. Model evaluation under practicalreal-world scenarios is also rarely considered—e.g., public areas where subjects appear in both training and validation, or restricted environments like military bases where reliable identification is critical. Furthermore, the existing gait datasets are not designed for spoofing detection tasks. CASIA-B was originally designed to test robustness against natural variability rather than spoofing. However, given the lack of dedicated visual spoofing datasets, BG and CL sequences serve as spoofing analogs, allowing assessment of deep temporal networks' sensitivity to deliberate silhouette alterations.

To address these limitations and advance the state of gait anti-spoofing, this work undertakes a systematic comparison of advanced deep learning architectures. We investigate the efficacy of models incorporating Long Short-Term Memory (LSTM), Gated Recurrent Unit (GRU), and a Mamba-inspired architecture for their ability to detect spoofing attacks when processing sequences of spatial features extracted by a common ResNet-based CNN backbone. The main contributions of this paper to the field of gait anti-spoofing are:

- The establishment of a reliable and repurposed CASIA-B dataset baseline specifically for the gait anti-spoofing task. We also introduce the first application of ecologically valid evaluation strategies to PAD, creating of two complex operational conditionsreal-world application scenarios using the available datasets by varying the method of data splitting for training and validation. The first scenario targets public areas that are frequently visited, where the visitor may appear partially in both the training and validation datasets at the same time but in different positions; thus, random splitting was used. On the other hand, the second scenario represents restricted areas that are not authorized for general access. For this case, we used LNSOCV for the first time in gait anti-spoofing, with 20% of the dataset used for validation in each fold.
- The first systematic comparison of advanced temporal architectures applied specifically to gait PAD—a task categorically different from general gait recognition. We created multi-architecture models, starting with initial models that achieved moderate results, and finalized three models achieving excellent outcomes. These models used LSTM, GRU, and the official Mamba selective state space model. A comprehensive comparative evaluation of these architectures was performed specifically for robust gait spoofing detection.
- Demonstration that these advanced temporal models significantly outperform baseline configurations in distinguishing bona fide gaits from spoofing attacks, with the identification of a GRU-based architecture as the top-performing model for open-access gait anti-spoofing, achieving state-of-the-art results, while demonstrating that LSTM-based architectures maximize effectiveness in restricted-access conditions.
- An in-depth analysis of the evaluated models, focusing on their effectiveness in spoofing detection, their generalization capabilities against attacks, and the impact of architectural choices on anti-spoofing performance across different deployment conditions. This includes theoretical justification for why the GRU's simpler gating suffices in general-access scenarios, whereas the LSTM's full memory cell is necessary for subject-disjoint generalization.

To ensure the reproducibility of the reported results, the source code used in this study have been made publicly available at: https://github.com/stars-of-orion/GaitSpoofNet The remainder of this paper is organized as follows: Section 2 reviews the foundational framework of gait recognition and related work in anti-spoofing. Section 3 details the proposed architectures and data partitioning strategies evaluated for spoofing detection. Section 4 presents the experimental setup and comparative anti-spoofing results across different operational scenarios. Section 5 discusses the findings in the context of practical gait security, and Section 6 concludes the paper. Furthermore, the detailed theoretical formulations and state-space mechanics of the advanced temporal sequence models (LSTM, GRU, and Mamba) are provided in Appendix 1.

## 2 Related work

### 2.1 Gait recognition and anti-spoofing

Because biometric recognition technology uses human behavioral traits to confirm an individual's identity, it is becoming increasingly popular in both academia and industry. There are many identification techniques, some of the most widely used ones being voice recognition, facial recognition, iris scanning, and so on. All of these techniques, however, share the flaw of only being able to identify a person at close range. In contrast, gait recognition outperforms alternative modalities when identifying individuals from a significant distance. This process works by isolating the distinct spatiotemporal patterns of a person's walk. For security and surveillance systems, this biometric modality provides a number of clear operational benefits. Above all, it can be observed from a distance without the subject's direct participation. Furthermore, a person's normal gait is so deeply ingrained that it is very challenging to totally hide or change it without arousing suspicion.

**Figure 1:**
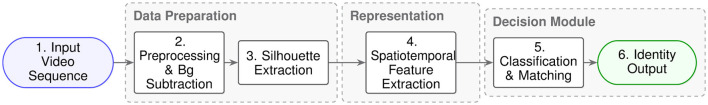
General framework of a vision-based gait recognition system.

Gait representation has evolved across several paradigms (Figure 1). Early work used handcrafted descriptors such as the Gait Energy Image (GEI) (Han and Bhanu, 2006), which averages silhouettes over a complete walking cycle. Deep learning later replaced these with CNNs (Chao et al., 2019) for frame-level spatial extraction, subsequently combined with RNNs to capture temporal evolution across the gait cycle.

Within the broader framework of gait recognition, this paper focuses on PAD problem for vision-based, silhouette-reliant systems. This narrows the focus from general recognition to the relatively unexplored field of gait anti-spoofing. The core threat model under consideration is the appearance-based attack. In this scenario, an adversary attempts to fool the system by actively modifying their natural silhouette, usually through clever clothing changes or the addition of carried objects. This vulnerability is conceptually similar to presentation attacks in facial recognition systems, where attackers use physical artifacts such as printed images or silicone masks to modify the visual appearance presented to a camera sensor.

### 2.2 Gait presentation attack detection (PAD)

#### 2.2.1 Overview of gait spoofing

Gait recognition has been extensively studied (Shen et al., 2025). PAD is a critical research area across biometrics—face (Li et al., 2024), fingerprint (Zhang Y. et al., 2024)—and gait is no exception, with attacks including impersonation and video replay. Early deep learning for gait used CNNs for frame-level features, sometimes with RNNs for sequence modeling. Gait spoofing research falls into four categories: sensor-based attacks, fake sequence generation, performance-based attacks, and computer-vision spoofing. The last category largely addresses theoretical concepts without deploying real models, underlining the need for stable vision-based anti-spoofing.

#### 2.2.2 Vision-based gait spoofing

We begin with the first category: gait spoofing in computer vision. This topic has been explored in a limited number of studies, primarily by recurring authors who often present similar concepts. A series of studies by Hadid et al. established the foundational evidence for appearance-based gait spoofing, demonstrating that deliberate clothing impersonation especially when the attacker shares a similar body shape with the victim can successfully deceive silhouette-based biometric systems, and evaluated two state-of-the-art recognition systems against such attacks (Hadid et al., 2012, 2013, 2015). Bustard et al. (2014) examined two spoofing strategies wearing identical clothing and selecting a morphologically similar victim- finding that both attack types undermine current gait systems. Masood and Farooq (2017) investigated how garment replication and physical build affect classification outcomes, and proposed testing protocols specific to the gait spoofing threat.

#### 2.2.3 Sensor data and wearable attacks

In Boulgouris et al. (2006), time-normalized gait cycles measured by a hip-mounted three- axis accelerometer are used for authentication. Gafurov et al. (2007) analyzed minimal-effort impersonation and closest-person attacks using hip-accelerometer data from 100 participants across 760 sequences. Mjaaland et al. (2011) collected wearable acceleration data and used regression analysis to assess whether subjects improve their gait-imitation ability over time. Kumar et al. (2015) demonstrated that a digital treadmill can replicate smartphone-recorded stride patterns, evaluating the resulting GBAS attack on 18 users. Shrestha et al. (2016) introduced WUZIA, a multi-sensor Zero-Interaction Authentication system shown to resist active gait-replication attacks across multiple devices. Zhu et al. (2021) proposed the “one cycle attack”, using K-means clustering to identify adversarial gait cycles capable of circumventing six state-of-the-art models. Kumar et al. (2021) investigated treadmill-assisted spoofing on wearable sensor-based authentication under realistic deployment conditions. In Song et al. (2023), a security analysis of wearable gait systems revealed counterfeiting vulnerabilities, prompting the Pistis authentication protocol combining gait biometrics with liveness detection. Recent research has built on these fundamental contributions to meet the urgent need for robust sensor-based comparisons. By utilizing high-frequency sampling of inertial data to capture minute gait nuances, researchers have developed a unified local-global feature extraction network for human gait detection using smartphone sensors (Das et al., 2022). Recent architectures for sensor-based gait recognition employ ensemble networks unified with distinct subnetworks to further improve classification stability across various settings (Das et al., 2024b).

### 2.3 Performance-based spoofing attacks

In the third category, studies attempt to compromise gait recognition systems, demonstrating how gait can be spoofed through performance-based attacks. Tieu et al. (2017) developed a CNN-based gait anonymization technique that adds a learned “noise gait” to the original sequence, preventing recognition while preserving visual naturalness. Jia et al. (2019) used a GAN to render mimicked walking video from a target scene image and a source subject's gait sequence. Guo et al. (2020) demonstrated black-box vulnerability of CNN-based gait classifiers to Fast Gradient Sign Method (FGSM) adversarial attacks, highlighting reliance on shadow-model quality. Bukhari et al. (2022) explored CNN-based gait detection vulnerabilities under clothing, object-carrying, and speed variations. Hirose et al. (2023) attempted to synthesize fake gait silhouettes from a single photo, finding that a single-frame feature vector does not fully capture the target's walking characteristics.

### 2.4 Methodologies in gait recognition on the CASIA-B dataset

#### 2.4.1 Spatio-temporal modeling on the CASIA-B benchmark

This study focused on gait spoofing detection, and one of the core problems that inspired us was the lack of an available dataset for gait spoofing, so we reused the well-known CASIA B (Yu et al., 2006) dataset, which is commonly used in gait recognition. Further research is needed to identify and discuss prior gait spoofing detection studies that have specifically utilized the same dataset employed in this work to provide a direct comparative context within the literature. Our study aims to contribute a robust baseline on this dataset for future anti-spoofing research using advanced temporal models. More sophisticated temporal models like LSTMs (Graves, 2012) and GRUs (Cho et al., 2014) have shown superior performance in capturing long-range dependencies critical for analyzing complex sequences. Recently, state-space models (SSMs) like Mamba Gu and Dao (2023) have emerged as powerful alternatives for efficient long-sequence modeling. A systematic comparison of these diverse advanced architectures for their efficacy in robust gait spoofing detection is the core focus of this study.

We categorized the existing gait recognition research based on the input representation used in gait detection models—whether silhouette-based, pose-based, or a combination of both. However, in our work, we mainly focus on silhouette-based approaches, specifically those that use the CASIA B dataset.To establish a comprehensive benchmarking standard for these approaches, Fan et al. (2023) developed OpenGait, a flexible codebase that rigorously re-evaluates existing methods to introduce GaitBase. This structurally simple but empirically powerful baseline demonstrates consistent, robust performance across multiple public datasets in both indoor and outdoor scenarios, emphasizing the critical need for practical generalization in gait recognition. Castro et al. (2024) presented AttenGait, employing trainable attention mechanisms over arbitrary input modalities, including optical flow, for state-of-the-art gait identification. Song et al. (2025) combined a Multi-scale Dilated Temporal Extractor with a View-aware Part-wise Attention mechanism to improve view-robust gait representation. Deng et al. (2025) introduced the LFHEI representation using local optical flow histograms and deep metric learning for frontal-view gait recognition. Kumar et al. (2024) proposed BGaitR-Net, combining a Convolutional Variational Autoencoder with Bi-LSTM to reconstruct occluded gait sequences. Because visual occlusion degrades data quality, signal integrity remains a significant challenge in this field. Drawing inspiration from broader biometric methodologies to address this, adaptive threshold-based gait authentication systems incorporating quality measures have been developed (Das et al., 2024a), enabling the system to dynamically modify its sensitivity based on the reliability of the recorded sequence. Qiao et al. (2025) applied decoupled spatial-temporal low-rank convolutions with a convolution block attention module for cross-view gait identification. P and Poornachandran (2025) combined ConViT with sparse edge-based feature extraction and random convolutions for GEI-based person recognition. Kumar et al. (2025) used selective search with L-softmax and ResNet-V1 for attribute-aware gait region classification. Zhou et al. (2025) addressed cross-domain generalization via focused data distillation and Domain-Specific Batch Normalization. In Mogan et al. (2024), an ensemble of DenseNet-201, VGG-16, and ViT is fused with a window-GEI representation for gait identification. Avais Hanif et al. (2023) extracted optical-flow motion zones and fused raw-frame and flow-based features using a normal-distribution-based scheme. Hua et al. (2024) proposed TMFL, combining multi-scale inter-frame motion extraction with temporal soft-attention aggregation. Similarly addressing the demand for robust spatiotemporal feature extraction, Lin et al. (2021) introduced a Global and Local Feature Extractor (GLFE) combined with Local Temporal Aggregation (LTA). This framework seamlessly integrates global appearances and local region details while reducing temporal resolution to achieve higher spatial fidelity, thereby significantly enhancing the discriminative power of the visual representations. Zhai et al. (2025) proposed a global-local spatiotemporal network with multi-resolution feature extraction and multi-branch fusion. Hasan et al. (2024) achieved 97.17% accuracy using pixel-level CNN classification (VGG16, VGG19, NASNet, EfficientNetB0, Xception) on CASIA-B silhouettes. Yousef et al. (2023) combined GAN-based gait image generation with PSO/GWO feature selection and SMOTE balancing for DNN-based recognition. Pioneering the part-based approach for spatio-temporal modeling, Fan et al. (2020) proposed GaitPart. Because different body parts exhibit distinct visual and movement patterns, they utilized a Focal Convolution Layer for fine-grained spatial learning and a Micro-motion Capture Module (MCM) to extract short-range temporal features from predefined body segments. Ultimately, this localized strategy yielded state-of-the-art results on the CASIA-B dataset. Yaprak and Gedikli (2025) applied ensemble CNN learning across five horizontal GEI body-part segments for part-based gait recognition. Jain et al. (2024) presented SMD-CCDN, combining B-Spline Magnitude Disparity deformation registration with Cross-Correlated LSTM recognition. Xi et al. (2024) proposed a self-supervised semi-supervised framework for spatiotemporal gait representations. Pan et al. (2024) proposed GaitLRDF, using a 2D convolution formulation to produce discriminative feature representations. Deng et al. (2024) fused dense optical flow with holistic frontal-view silhouettes for multi-modal gait recognition. Meng et al. (2023) enhanced recognition using 3D human body reconstruction via an HMR module to generate compact, redundancy-free gait representations. Anusha and Sunil (2024) used contour vertices for frontal gait feature extraction. Ray et al. (2024) fused skeleton data from multiple top-down and bottom-up pose estimators in a multi-biometric gait framework. Aman et al. (2024) compared four deep learning models on CASIA-B, with CNN achieving 97.12% accuracy. Ammar Alsherfawi Aljazaerly et al. (2025) synthesized high-quality gait silhouettes from arbitrary views using a 3D human deformation model.

#### 2.4.2 Advanced feature extraction: multi-scale, attention, and ensembles

Recent advancements in gait recognition have increasingly depended on multi-scale feature extraction, attention mechanisms, and hybrid architectures to improve model robustness. Multi-scale modeling enables networks to capture both fine-grained local anomalies, such as the rigid boundaries of a carried bag, and broader dynamics, such as an altered walking stride (Zhang Z. et al., 2024). Additionally, both spatial and temporal attention mechanisms have become common for dynamically weighting the most important body parts or time frames. This helps the model ignore noisy data from clothing variations (Sheng and Li, 2021). To boost generalization, recent studies often use hybrid deep learning models, combining different architectures such as CNNs, Vision Transformers (ViTs), and Recurrent Networks into unified pipelines (Burges et al., 2024). This combination of focused attention, scale variation, and architectural diversity creates a highly robust feature space. Motivated by these findings, our proposed architectures combine convolutional backbones with temporal and attention modules to effectively separate spoofing artifacts.

## 3 Methodology: architectures for gait spoofing detection

**Figure 2:**
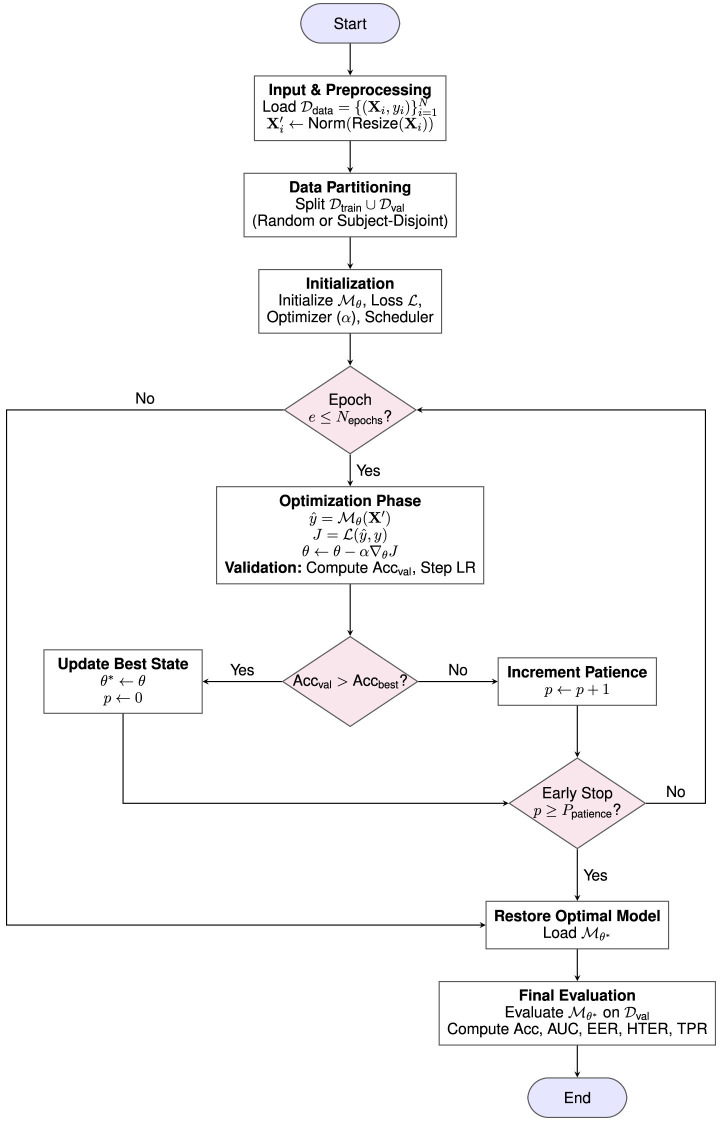
Mathematical workflow of the GaitSpoofNet training, optimization, and evaluation pipeline.

Securing biometric systems against spoofing is a growing priority as gait recognition gains traction. The field addresses this unevenly: sensor-based approaches have reached considerable maturity, while camera-based systems severely lack practical PAD defenses–most literature still treats vision-based spoofing as theoretical rather than an immediate threat. To address this gap, the primary objective is to build a dependable, vision-based architecture for detecting gait spoofing in operational conditions. At the same time, no recent study has attempted to evaluate gait spoofing models based on complex operational conditionsreal-world application scenarios. The second objective is to evaluate the proposed models under two complex operational conditionsreal-world operational scenarios: a general-access environment and a restricted, unauthorized-access environment. We developed eight models called (GaitSpoofNet-A, GaitSpoofNet-B, GaitSpoofNet-C, GaitSpoofNet-D, GaitSpoofNet-E, GaitSpoofNet-F, GaitSpoofNet-G, and GaitSpoofNet-H) and classified these models into two categories (Group A and Group B). The overall workflow of the proposed models is illustrated in Figure 2, and the training and evaluation procedure is detailed in Algorithm 1. To extract spatial gait features for spoofing detection, the GaitSpoofNet models (A-G), as summarized in Table 1, evaluate various CNN backbone topologies. Models D, E, F, and G provide two backbone options: a customized CNN and a modified ResNet18. GaitSpoofNet-A, GaitSpoofNet-B, and GaitSpoofNet-C use exclusively custom CNN backbones. In the custom CNN versions, these models used four convolutional layers with 3 × 3 kernels, ReLU activation, batch normalization, and 2 × 2 max pooling after each layer. Their filter progression follows a consistent pattern from 1 to 64, 128, 256, and up to 256 output channels. Conversely, GaitSpoofNet-A and GaitSpoofNet-B rely exclusively on deeper 8-layer customized CNNs. In the ResNet18-based structures, the network produces a 512-dimensional embedding after the first convolution layer is modified to take 1-channel (grayscale) input.

Algorithm 1 Gait spoofing detection training and evaluation procedure. 
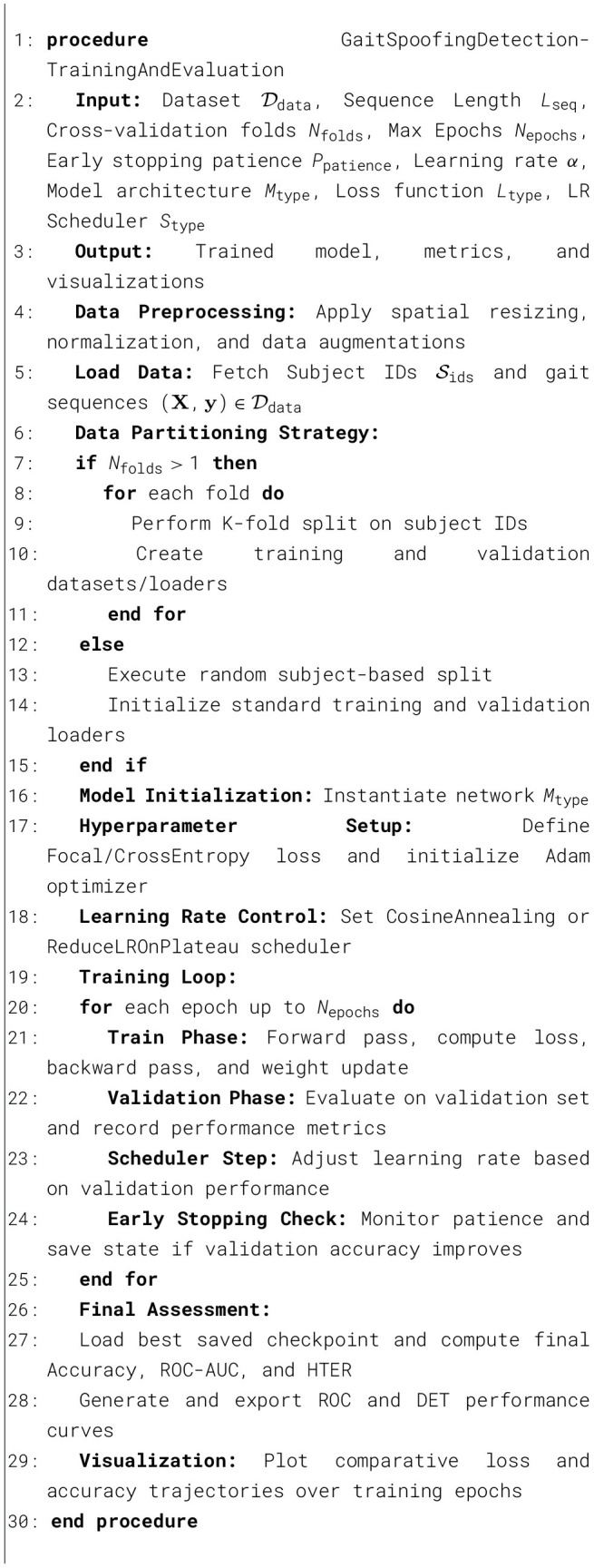

**Table 1:** Comparative CNN architectures of GaitSpoofNet variants (A–H).

Model	Backbone options	# Conv layers	Filter progression	Pooling + output
GaitSpoofNet-A	Custom	8	1 → (64 × 2) → (128 × 2) → (256 × 2) → (512 × 2)	MaxPool after 2nd, 4th, 6th + AdvAvgPool Output: 512
GaitSpoofNet-B	Custom	8	1 → (64 × 2) → (128 × 2) → (256 × 2) → (512 × 2)	MaxPool after 2nd, 4th, 6th + AdvAvgPool Output: 512
GaitSpoofNet-C	Custom	5	1 → 64 → 128 → 256 → 512 → 512	MaxPool after each conv Output Channels: 512
GaitSpoofNet-D	Custom / ResNet-18	4 (Custom)	1 → 64 → 128 → 256 → 256	MaxPool after each conv Output Channels: 256 (Custom) / 512 (ResNet)
GaitSpoofNet-E	Custom / ResNet-18	4 (Custom)	1 → 64 → 128 → 256 → 256	MaxPool after each conv Output Channels: 256 (Custom) / 512 (ResNet)
GaitSpoofNet-F	Custom / ResNet-18	4 (Custom)	1 → 64 → 128 → 256 → 256	MaxPool after each conv Output Channels: 256 (Custom) / 512 (ResNet)
GaitSpoofNet-G	Custom / ResNet-18	4 (Custom)	1 → 64 → 128 → 256 → 256	MaxPool after each conv Output Channels: 256 (Custom) / 512 (ResNet)
GaitSpoofNet-H	Custom / ResNet-18	4 (Custom)	1 → 64 → 128 → 256 → 256	MaxPool after each conv Output Channels: 256 (Custom) / 512 (ResNet)

In this section we describe our approach used in gait spoofing detection including how to Preprocess the current famous dataset (CASIA B) in gait detection to use in gait spoofing and the model architectures used, beside the two training methods that represent the two real world application scenario and evaluation metrics.

### 3.1 Dataset

The study utilized CASIA B, a large multiview gait database collected in January 2005 and a typical dataset in gait recognition. Eleven views were used to record the gait data for the 124 participants. Three variations are taken into consideration independently: changes in carrying condition, clothes, and view angle. For this work, gait sequences are categorized into two classes: normal and spoofed. Normal refers to “NM” walking condition. The spoofed class corresponds to the “BG” and “CL” walking conditions that simulate spoofing attempts in gait recognition. In this study, it is assumed that wearing jackets and carrying bags are prohibited in normal samples of both scenarios. This constraint was explicitly applied to establish a controlled baseline for evaluating the core spatio-temporal architectures. By excluding severe silhouette occlusions and shape-altering covariates, we ensure that the models are evaluated strictly on their baseline ability to capture pure human locomotion and temporal gait dynamics, rather than their robustness to heavy occlusions. Addressing these complex environmental covariates remains a vital direction for future research. In this study, gait spoofing is defined as the deliberate alteration of an individual's appearance, such as wearing different clothes or carrying a bag, to deceive a gait-based authentication system. Building on existing literature regarding gait anti-spoofing (Hadid et al., 2012, 2013, 2015; Bustard et al., 2014; Masood and Farooq, 2017), we classify this as an appearance-based Presentation Attack (PA). The key difference between a malicious PA and a benign variation is the intent to deceive. For instance, in a practical security setting, an attacker who understands that the system relies on silhouettes might intentionally wear a bulky coat to avoid detection.

Although CASIA-B was originally created for covariate-based gait recognition rather than PAD, its use in this study is both necessary and well-supported by existing literature. This choice is driven by three main factors. First, there is currently no dedicated, publicly available silhouette-based gait dataset designed specifically for PAD. Consequently, current vision-based gait PA research (Hadid et al., 2012, 2013; Bustard et al., 2014; Hadid et al., 2015; Hirose et al., 2023) often relies on repurposing CASIA-B or generating synthetic silhouettes. Second, utilizing this dataset allows us to follow established practices, enabling direct comparisons with previous methods while we incorporate deep temporal models under a strict subject-disjoint cross-validation protocol. Finally, CASIA-B is well-suited for simulating appearance-based attacks. By offering three different walking conditions (NM, BG, and CL) for the same 124 subjects across 11 viewing angles, it allows for the controlled binary classification (normal vs. appearance-altered) needed to assess PAD models under structured variations.

### 3.2 Data preprocessing and augmentation

First, all gait sequences are processed frame by frame. Each frame is converted to grayscale for all models.

#### 3.2.1 Preprocessing for Group A (GaitSpoofNet-A, GaitSpoofNet-B, and GaitSpoofNet-C)

The dataset preprocessing includes three operations: resizing, augmentation, and normalization. The first preprocessing step resizes each frame before it is used as model input. In the GaitSpoofNet-A and GaitSpoofNet-B models, we resized the frames to 64 × 64 pixels, and in the GaitSpoofNet-C model, we resized the frames to 96 × 96 pixels. The next step is augmentation. Data augmentation techniques included random horizontal flip, random affine transformations (including translation, scaling, and shear), and random erasing. The final step is the normalization of pixel values.

#### 3.2.2 Preprocessing for Group B (GaitSpoofNet-D, GaitSpoofNet-E, GaitSpoofNet-F, GaitSpoofNet-G and GaitSpoofNet-H)

For Group B, we resize each frame to 96 × 96 pixels and normalize the pixel values with a mean of 0.5 and a standard deviation of 0.5. The models applied data augmentation to the training dataset, including random brightness and contrast adjustments, Gaussian noise addition, horizontal flipping, as well as shift, scale, and rotation transformations. At the same time, coarse dropout was used to simulate occlusions and enhance robustness.

### 3.3 Model architectures

#### 3.3.1 Architectures of Group A

The models in this group use a custom CNN backbone. GaitSpoofNet-A and GaitSpoofNet-B employ deeper architectures with 512 output channels, feeding the extracted features into a recurrent temporal module, while GaitSpoofNet-C used a customized CNN with 512 output channels and more layers. This group employs two-layer LSTM models for temporal modeling, with dropout rates of 0.4 for GaitSpoofNet-A and GaitSpoofNet-B, and 0.3 for GaitSpoofNet-C. GaitSpoofNet-C utilized a unidirectional LSTM with a hidden size of 1024, while GaitSpoofNet-A and GaitSpoofNet-B utilized a bidirectional LSTM with a hidden size of 256 per direction (512 concatenated). All models implement the attention mechanism using a single linear layer. Finally, each model incorporated a fully connected layer for binary classification. Table 2 shows the architecture settings for each model.

**Table 2:** Architectures of Group A models including backbone and temporal configurations.

Feature	GaitSpoofNet-A	GaitSpoofNet-B	GaitSpoofNet-C
CNN Backbone	Custom (8-Layer)	Custom (8-Layer)	Custom (5-Layer)
Temporal Model	2-Layer BiLSTM (512)	2-Layer BiLSTM (512)	2-Layer UniLSTM (1024)
Attention Mechanism	Single Linear Layer	Single Linear Layer	Single Linear Layer
Sequence Sampling	Random single sequence	Random single sequence	Random single sequence
Loss Function	CrossEntropy with Smoothing	CrossEntropyLoss	CrossEntropyLoss
Scheduler	CosineAnnealingLR	ReduceLROnPlateau	ReduceLROnPlateau
Early Stopping	Validation Accuracy	Validation Accuracy	Validation Accuracy

#### 3.3.2 Architectures of Group B

The group B includes GaitSpoofNet-D, GaitSpoofNet-E, GaitSpoofNet-F, GaitSpoofNet-G and GaitSpoofNet-H. The models follow a two-stage architecture and utilize a ResNet18 backbone pre-trained on ImageNet as the primary spatial feature extractor. The first convolutional layer was modified to accept single-channel (grayscale) input. A 512-dimensional feature vector is extracted from the global average pooling layer. Table 3 shows the architecture settings for each model.

**Table 3:** Architectures of Group B models including backbone and temporal configurations.

Feature	GSN-D	GSN-E	GSN-F	GSN-G	GSN-H (inspired Mamba)
CNN Backbone	Custom/ResNet-18	Custom/ResNet-18	Custom/ResNet-18	Custom/ResNet-18	ResNet-18
Temporal Model	1-Layer BiGRU (256)	2-Layer Mamba SSM (512)	1-Layer BiGRU (256)	1-Layer BiLSTM (256)	2-Layer Inspired Mamba (512)
Attention	Single Linear Layer	Global Avg Pooling	Single Linear Layer	Single Linear Layer	Global Avg Pooling
Sampling	Overlapping stride	Overlapping stride	Overlapping stride	Overlapping stride	Overlapping stride
Loss Function	CrossEntropyLoss	CrossEntropyLoss	CrossEntropyLoss	CrossEntropyLoss	CrossEntropyLoss
Scheduler	CosineAnnealingLR	CosineAnnealingLR	CosineAnnealingLR	CosineAnnealingLR	CosineAnnealingLR
Early Stopping	Val Accuracy	Val Accuracy	Val Accuracy	Val Accuracy	Val Accuracy

To precisely define how spatial features are temporally aggregated, the mathematical formulations for our top-performing Group B models are detailed below.

##### 3.3.2.1 GaitSpoofNet-F: bidirectional GRU with CNN and attention

Let $x_{t}^{\left(i\right)}\in {\mathbb{R}}^{1\times H\times W}$ be frame *t* of silhouette sequence *i*. The GaitSpoofNet-F model processes each frame through the ResNet18 backbone to obtain a 512-dimensional feature vector:


$$\begin{array}{l} f_{t}=\text{ResNet18\_GAP}\left(x_{t}\right)\in {\mathbb{R}}^{512}\;\left(GS1\right) \end{array}$$

A single-layer bidirectional GRU models the temporal dynamics of the silhouette sequence. Building upon the standard GRU update equations detailed in Appendix 1.2, the forward and backward passes are computed as:


$$\begin{array}{lll} h_{t}^{\to } & = & \text{GRU\_fwd}\left(f_{t},h_{t-1}^{\to }\right)\in {\mathbb{R}}^{256}\;\left(GS2\right)\\ h_{t}^{\leftarrow } & = & \text{GRU\_bwd}\left(f_{t},h_{t+1}^{\leftarrow }\right)\in {\mathbb{R}}^{256}\;\left(GS3\right)\\ h_{t} & = & \left[h_{t}^{\to }||h_{t}^{\leftarrow }\right]\in {\mathbb{R}}^{512}\;\left(GS4\right) \end{array}$$

A learned soft-attention mechanism then weights each time step by its discriminative importance. This produces a context vector *c* that is independent of frame order and robust to variable-length sequences:


$$\begin{array}{lll} e_{t} & = & W_{a}\cdot h_{t}+b_{a}\in {\mathbb{R}}^{1}\;\left(GS5\right)\\ \alpha_{t} & = & \frac{\exp\left(e_{t}\right)}{\sum_{\tau }\exp\left(e_{\tau }\right)}\in \mathbb{R}\;\left(GS6\right)\\ c & = & \underset{t}{\sum }\alpha_{t}\cdot h_{t}\in {\mathbb{R}}^{512}\;\left(GS7\right) \end{array}$$

Finally, the classifier applies dropout and a fully connected layer to produce binary (bona fide / attack) logits:


$$\begin{array}{l} ŷ=\text{FC}\left(\text{Dropout}\left(c\right)\right)\in {\mathbb{R}}^{2}\;\left(GS8\right) \end{array}$$

Compared to a vanilla GRU applied to raw pixels, GaitSpoofNet-F differs by extracting rich spatial appearance features prior to temporal modeling, capturing both forward and backward temporal context, and utilizing interpretable frame-level importance weights to focus on the most discriminative gait cycles.

##### 3.3.2.2 GaitSpoofNet-G: bidirectional LSTM with CNN and attention

GaitSpoofNet-G follows the same CNN and Attention pipeline but substitutes the GRU with an LSTM cell. Building upon the standard LSTM foundations in Appendix 1.1, the forward pass replaces equations GS2-GS4 with:


$$\begin{array}{lll} f_{t} & = & \text{ResNet18\_GAP}\left(x_{t}\right)\in {\mathbb{R}}^{512}\;\left(LS1\right)\\ \left(h_{t}^{\to },c_{t}^{\to }\right) & = & \text{LSTM\_fwd}\left(f_{t},h_{t-1}^{\to },c_{t-1}^{\to }\right)\in {\mathbb{R}}^{256}\;\left(LS2\right)\\ \left(h_{t}^{\leftarrow },c_{t}^{\leftarrow }\right) & = & \text{LSTM\_bwd}\left(f_{t},h_{t+1}^{\leftarrow },c_{t+1}^{\leftarrow }\right)\in {\mathbb{R}}^{256}\;\left(LS3\right)\\ h_{t} & = & \left[h_{t}^{\to }||h_{t}^{\leftarrow }\right]\in {\mathbb{R}}^{512}\;\left(LS4\right) \end{array}$$

The attention mechanism and classifier remain identical to equations GS5-GS8. The key architectural addition is the cell state *c*_*t*_, which provides an explicit long-range memory channel separate from the hidden state *h*_*t*_. This allows the model to maintain inter-cycle temporal context across the full 20-frame window. This explicit memory is particularly beneficial under the LNSOCV evaluation protocol, where the model must generalize to entirely unseen subjects. In contrast to GaitSpoofNet-F, GaitSpoofNet-G has approximately 33% more parameters in the temporal module but achieves a lower Equal Error Rate (EER) in subject-disjoint scenarios, confirming that the richer memory mechanism confers a generalizability advantage.

##### 3.3.2.3 GaitSpoofNet-E: official selective state space model (Mamba)

GaitSpoofNet-E replaces the recurrent temporal module with the official Mamba selective state space model (mamba-ssm). Unlike structurally-inspired prototypes, this implementation includes the complete selective scan detailed in Appendix 1.3. The time-step Δ_*t*_ and projection matrices *B*_*t*_ and *C*_*t*_ are computed as input-dependent functions of the current frame feature *f*_*t*_, enabling the model to dynamically decide which temporal context to retain or discard.

Each OfficialMambaBlock adds a pre-LayerNorm and residual connection to stabilize training:


$$\begin{array}{lll} x\text{\_}norm_{t} & = & \text{LayerNorm}\left(x_{t}\right)\;\left(MB1\right)\\ s_{t} & = & \text{MambaSSM}\left(x\text{\_}norm_{t}\right)\;\left(MB2\right)\\ y_{t} & = & x_{t}+\text{Dropout}\left(s_{t}\right)\;\left(MB3\right) \end{array}$$

A stack of *K* = 2 such blocks forms the OfficialSequentialMamba. The full GaitSpoofNet-E forward pass processes the 20-frame sequence as follows:


$$\begin{array}{lll} f_{t} & = & \text{ResNet18\_GAP}\left(x_{t}\right)\in {\mathbb{R}}^{512}\;\left(ME1\right)\\ F & = & \left\{f_{1},\ldots ,f_{L}\right\}\in {\mathbb{R}}^{L\times 512}\;\left(ME2\right)\\ M_{\text{input}} & = & {\text{Linear}}_{\text{proj}}\left(F\right)\in {\mathbb{R}}^{L\times 512}\;\left(ME3\right)\\ O & = & \text{OfficialSequentialMamba}\left(M_{\text{input}}\right)\in {\mathbb{R}}^{L\times 512}\;\left(ME4\right) \end{array}$$

Global average pooling over the sequence dimension aggregates temporal information before the binary classifier:


$$\begin{array}{lll} c & = & \frac{1}{L}\underset{t}{\sum }O_{t}\in {\mathbb{R}}^{512}\;\left(ME5\right)\\ ŷ & = & \text{FC}\left(\text{Dropout}\left(c\right)\right)\in {\mathbb{R}}^{2}\;\left(ME6\right) \end{array}$$

This approach is fundamentally different from LSTM and GRU architectures. The state update is implemented as a hardware-efficient parallel scan rather than sequential recurrence, yielding O(L) time and memory complexity, and the selectivity arises directly from the input-dependent parameterization of the SSM matrices rather than a separate gate network.

##### 3.3.2.4 GaitSpoofNet-H: inspired Mamba implementation

GaitSpoofNet-H provides a comparative baseline to the official Mamba model by using a modified Sequential Mamba architecture. It operates on the same ResNet-18 backbone $f_{t}\in {\mathbb{R}}^{512}$ and features two inspired Mamba blocks with a hidden dimension of 512 and a state dimension of 16. The main structural difference between this inspired version and the official Mamba (GaitSpoofNet-E) is the addition of an 8-head projection mechanism in the state-space formulation. While the official Mamba uses a single continuous state projection without attention-like heads, GaitSpoofNet-H connects state-space models with multi-head mechanisms. Unlike the LSTM and GRU models, which depend on learned soft attention, GaitSpoofNet-H gathers temporal features through global average pooling before the classification layer. With about 17.3 million parameters, it provides a higher-capacity, structurally different alternative to the official Mamba implementation.

### 3.4 The training and data splitting strategy for practical applications

While no two evaluation protocols can capture the entirety of practical environmental variability, we designed our data splitting strategy to simulate the two foundational operational extremes of biometric deployment. By evaluating the models under both general-access and restricted-access conditions, we test their scalability from environments where subjects are familiar to zero-trust environments where subjects are entirely unseen.

#### 3.4.1 General-access scenario (e.g., institutions)

This scenario represents environments in which individuals, such as staff members, may appear multiple times in recorded data, so, in training, it is acceptable for an individual to be partially repeated in both the training and validation data with different positions. This reflect practical operational settings in general-access environments. In this scenario we used random splitting that divides the dataset randomly into training and validation sets. This allows subjects to appear in both the training and validation sets under different conditions in the training set and validation set.

#### 3.4.2 Restricted-access scenario (e.g., military bases)

In contrast, the second scenario simulates restricted-access environments or places that work for detecting gait spoofing for anyone outside the institution staff, like military bases. So the training and validation sets must be different, and no subject can be repeated simultaneously in the training and validation datasets. To simulate this condition, we used LNSOCV strategy that prevents any subject from appearing in both. This subject-disjoint approach aligns with established best practices in vision-based gait analysis, where Leave-N-Subjects-Out protocols have been widely utilized to rigorously assess generalization and robustness against unseen identities (Makihara et al., 2012; Chao et al., 2019; Imoto et al., 2022).

### 3.5 Optimiser and training configuration

All Group B models use the AdamW optimiser with an initial learning rate η = 1 × 10^−4^ and weight decay λ = 0.01. AdamW was selected for three primary reasons:

1. **Decoupled weight-decay regularization:** as shown by Loshchilov and Hutter (2019), this reduces overfitting on small training subsets by applying the *L*_2_ penalty directly to the weights rather than to the gradient-adapted update.
2. **Adaptive learning rates:** the per-parameter learning rates accelerate convergence across the heterogeneous CNN and recurrent feature spaces.
3. **SSM fine-tuning superiority:** it has demonstrated empirical superiority over standard Adam specifically for State Space Model (SSM) fine-tuning.

The learning rate is annealed via CosineAnnealingLR (*T*_max_ = *N*_epochs_, η_min_ = η/100). This scheduler provides a smooth decay trajectory that prevents abrupt convergence stalling without requiring manual milestone scheduling.

### 3.6 Evaluation metrics

The performance of the proposed models was evaluated using several metrics, including:

**Accuracy** is defined as the ratio of correctly predicted samples to the total number of predictions, given by the Formula Equation 1:


$$\begin{array}{l} \text{Accuracy}=\frac{TP+TN}{TP+TN+FP+FN} \end{array}$$ (1)

where TP, TN, FP, and FN represent true positives, true negatives, false positives, and false negatives, respectively.

**ROC-AUC (Receiver Operating Characteristic—Area Under Curve)** evaluates the discriminative capability of the models, which calculate the trade-off between the true positive rate (TPR) and the false positive rate (FPR) across various thresholds.

Another key metric is **the Equal Error Rate (EER)**, which is the error rate at the operating point where the false acceptance rate (FAR) matches the false rejection rate (FRR). Lower EER values indicate better model performance during verification tasks.

Additionally, **APCER (Attack Presentation Classification Error Rate) and BPCER (Bona Fide Presentation Classification Error Rate)** are used to evaluate presentation attack detection. APCER is how often fake inputs are wrongly accepted as real, while BPCER is how often real inputs are wrongly rejected as fake.

**The Half Total Error Rate (HTER)** shows a balanced summary of both errors and is calculated as Equation 2:


$$\begin{array}{l} \text{HTER}=\frac{\text{APCER}+\text{BPCER}}{2} \end{array}$$ (2)

To further assess the study used other metrics such as Precision, Recall, confusion matrix, and F1-score.

## 4 Experiments and results for gait anti-spoofing

### 4.1 Comparative anti-spoofing performance

We start our experiments with reprocessing CASIA-B to be used in gait spoofing, so we relabeled CASIA-B as normal for subject condition nm (normal), and spoofed for subject conditions CL or BG. In the second step, we aimed to develop a stable model for gait spoofing. We start with random splitting that represents public places scenario. Starting with an initial model, we iteratively modified and enhanced the architecture while exploring various methods. All proposed models have linear computational complexity with respect to n, where n denotes the number of frames. This led to the development of eight models, labeled (GaitSpoofNet-A, GaitSpoofNet-B, GaitSpoofNet-C, GaitSpoofNet-D, GaitSpoofNet-E, GaitSpoofNet-F, GaitSpoofNet-G, and GaitSpoofNet-H), until we achieved the best performance with the final four models (GaitSpoofNet-E, GaitSpoofNet-F, GaitSpoofNet-G, and GaitSpoofNet-H). Based on the strong performance of these models, we considered a second practical evaluation scenario—the restricted area scenario—which is represented by LNSOCV. Accordingly, we divided our experiments into two parts: the first part is random splitting scenario tested on the eight models. We analyzed the performance of each model and how the results improved with each modification until reaching the best outcome. The second scenario involves LNSOCV, where we selected the top four models with the highest performance from the first scenario and evaluated them in a second practical evaluation representing a restricted area.

### 4.2 Scenario 1 (person-dependent)

**Figure 3:**
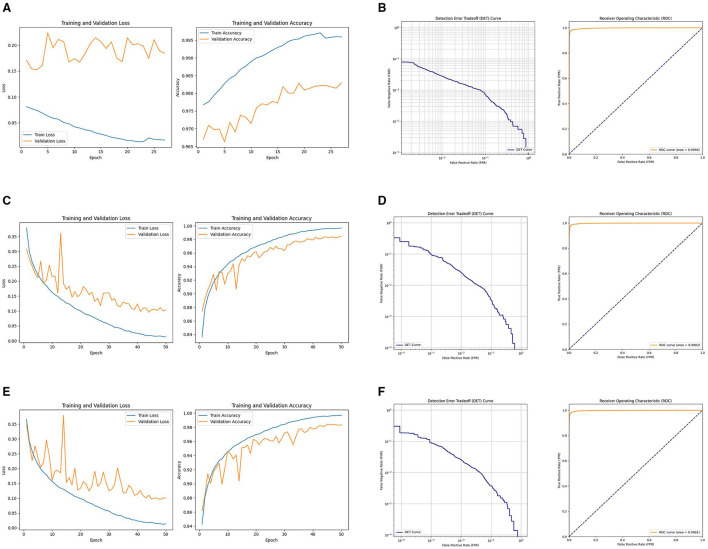
Comparison of model performance for GaitSpoofNet-E, F, and G under the random split scenario (Part 1). **(A, B)** Results for GaitSpoofNet-E. **(C, D)** Results for GaitSpoofNet-F, demonstrating faster convergence and a tighter train-validation accuracy gap indicating resistance to overfitting. **(E, F)** Detailed training trajectories and error trade-off curves for GaitSpoofNet-G. Note the slightly wider train-validation accuracy gap, indicating mild overfitting compared to the GRU architecture. **(A)** Training/Validation Curves - GaitSpoofNet-E. **(B)** DET & ROC - GaitSpoofNet-E. **(C)** Training/Validation Curves - GaitSpoofNet-F. **(D)** DET & ROC - GaitSpoofNet-F. **(E)** Training/Validation Curves - GaitSpoofNet-G. **(F)** DET & ROC - GaitSpoofNet-G.

In this scenario, to simulate typical operational setting for a public area, we first collected all subjects available in the dataset and saved them into one array. After that, we separated this array into normal and spoofed. For the training data, we split part of the normal samples and part of the spoofed samples and combined them to form the training dataset. The same method was used for the validation dataset. So, there is a probability that the same subject might appear partly in training and partly in validation. This represents public areas where the model might be trained with data from a subject and validated with data from the same subject but in a different condition. The GaitSpoofNet models evolved progressively from version A to H through improved data partitioning, refined training procedures, enhanced architectures, and more rigorous evaluation—culminating in strong generalization in versions E, F, G, and H. Table 4 shows the results for the first scenario, while Figure 3 illustrates the training and validation loss and accuracy, along with the ROC and DET curves for GaitSpoofNet-E, GaitSpoofNet-F, and GaitSpoofNet-G models.

**Table 4:** Full performance metrics for GaitSpoofNet Models A–H with random split (scenario 1).

Metrics	GSN-A	GSN-B	GSN-C	GSN-D	GSN-E (Off. Mamba)	GSN-F (GRU)	GSN-G (LSTM)	GSN-H (Insp. Mamba)
Accuracy	0.9349	0.9375	0.9304	0.9755	0.9830	0.9840	0.9839	0.9805
ROC-AUC	0.9756	0.9800	0.9727	0.9954	0.9966	0.9983	0.9981	0.9958
EER	0.0758	0.0660	0.0704	0.0280	0.0190	0.0165	0.0178	0.0214
APCER	0.1200	0.0358	0.1000	0.0405	0.0283	0.0233	0.0227	0.0330
BPCER	0.0284	0.1025	0.0545	0.0141	0.0097	0.0113	0.0118	0.0107
HTER	0.0742	0.0658	0.0698	0.0273	0.0190	0.0173	0.0173	0.0219

### 4.3 Scenario 2 (person-disjoint)

**Figure 4:**
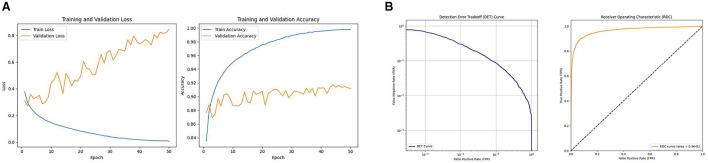
GaitSpoofNet-G model performance under LNSOCV scenario. **(a)** Training/validation curves—GaitSpoofNet-G. **(b)** DET & ROC—GaitSpoofNet-G.

In this scenario, we selected the top-performing models and changed the splitting strategy to LNSOCV. This scenario represents a restricted area where access is limited to authorized staff only. Therefore, we ensured that the complete frame sequences of each subject were included exclusively in either the training or validation set, without any overlap. The data was split into 80% training from one group of subjects and 20% validation from a different group. To ensure fairness, we applied 5-fold cross-validation, changing the validation subject IDs in each fold. Table 5 presents the performance of the models (E, F, G, and H) with LNSOCV. Visual results for LNSOCV for GaitSpoofNet-G model are also included, showing training and validation loss/accuracy curves, ROC curves, and DET curves (Figure 4).

**Table 5:** Full performance metrics for top models (E, F, G, H) for Scenario 2 (LNSOCV). Includes 95% confidence intervals derived from 5-fold variance.

Metrics	GaitSpoofNet-E (official mamba)	GaitSpoofNet-F (GRU)	GaitSpoofNet-G (LSTM)	GaitSpoofNet-H (inspired Mamba)
Avg. Accuracy	0.8900 ± 0.0165	0.8960 ± 0.0205	0.8986 ± 0.0177	0.8915 ± 0.0166
95% CI (Accuracy)	[0.8671, 0.9129]	[0.8676, 0.9244]	[0.8740, 0.9232]	[0.8685, 0.9145]
Avg. ROC-AUC	0.9434 ± 0.0130	0.9496 ± 0.0117	0.9477 ± 0.0126	0.9423 ± 0.0172
Avg. EER	0.1169 ± 0.0162	0.1109 ± 0.0170	0.1124 ± 0.0128	0.1165 ± 0.0168
Avg. APCER	0.1347 ± 0.0250	0.1248 ± 0.0280	0.1341 ± 0.0173	0.1426 ± 0.0453
Avg. BPCER	0.0941 ± 0.0292	0.0907 ± 0.0405	0.0803 ± 0.0337	0.0865 ± 0.0336
Avg. HTER	0.1144 ± 0.0149	0.1078 ± 0.0170	0.1072 ± 0.0141	0.1146 ± 0.0177
Avg. TPR @ 1% FPR	0.4158 ± 0.1759	0.5229 ± 0.1691	0.5287 ± 0.1989	0.4559 ± 0.2056

### 4.4 Architecture complexity and efficiency

For practical deployment, raw accuracy must be balanced with computational costs. Tables 6, 7 show the parameters, FLOPs, and inference delays for both evaluation scenarios. Notably, all top-performing Group B models run with very efficient forward passes (~6.4 GFLOPs) and low inference delays. GaitSpoofNet-G (LSTM) achieves the lowest overall inference delay (1.01 ms/sample), followed closely by GaitSpoofNet-E (Mamba SSM) at 1.06 ms/sample, in the LNSOCV scenario, showcasing the benefit of the hardware-efficient bidirectional scan at inference time, showcasing the benefits of hardware-aware parallelization in state-space models during inference when compared to sequential recurrent units. Despite the varying computational demands of these advanced architectures, their deployment ensures the highest accuracy and robustness for restricted-access environments.

**Table 6:** Computational complexity and inference efficiency of evaluated models (RS).

Model	Total parameters	GFLOPs	Inference latency (ms/sample)
GaitSpoofNet-A	15,193,283	25.4195	2.1966
GaitSpoofNet-B	15,193,283	25.4195	1.7114
GaitSpoofNet-C	35,389,315	19.0752	1.8330
GaitSpoofNet-D	12,354,499	6.4334	1.9347
GaitSpoofNet-E (Official Mamba)	14,825,410	6.4150	1.2972
GaitSpoofNet-F (GRU)	12,354,499	6.4334	1.3003
GaitSpoofNet-G (LSTM)	12,748,739	6.4413	1.3107
GaitSpoofNet-H (Insp. Mamba)	17,285,602	6.4991	2.1012

**Table 7:** Computational complexity and inference efficiency of top-performing models.

Model	Total parameters	GFLOPs	Inference latency (ms/sample)
GaitSpoofNet-E (Mamba SSM)	14,825,410	6.4150	1.0611
GaitSpoofNet-F (GRU)	12,354,499	6.4334	1.3465
GaitSpoofNet-G (LSTM)	12,748,739	6.4413	1.0085
GaitSpoofNet-H (Inspired Mamba)	17,285,602	6.4991	3.1119

### 4.5 Temporal dependency and feature separability

To measure the time needed for spoofing detection, an ablation study looked at sequence lengths from *T* = 1 to *T* = 100 frames (Tables 8, 9). Performance levels off at *T* = 20 frames for recurrent architectures, showing this is the best time window. A feature separability analysis (Table 10) also confirmed strong discriminative power, with the highest Fisher Discriminant Ratios (FDR) recorded at 1.797 for Mamba (open-access) and 1.391 for GRU (restricted-access).

**Table 8:** Temporal ablation study: impact of sequence length (T) on accuracy and EER (random split).

Model	T=1 (Acc / EER)	T=10 (Acc / EER)	T=20 (Acc / EER)	T=30 (Acc / EER)	T=50 (Acc / EER)	T=100 (Acc / EER)
GaitSpoofNet-A	0.8132 / 0.2031	0.8931 / 0.1191	0.9249 / 0.0824	**0.9305 / 0.0758**	0.9305 / 0.0758	0.9305 / 0.0758
GaitSpoofNet-B	0.7707 / 0.2363	0.8968 / 0.1141	0.9290 / 0.0746	**0.9419 / 0.0697**	0.9419 / 0.0697	0.9419 / 0.0697
GaitSpoofNet-C	0.7936 / 0.2185	0.8811 / 0.1222	0.9279 / 0.0833	**0.9384 / 0.0630**	0.9384 / 0.0630	0.9384 / 0.0630
GaitSpoofNet-D	0.8268 / 0.1753	0.9493 / 0.0526	**0.9839 / 0.0178**	0.9839 / 0.0178	0.9839 / 0.0178	0.9839 / 0.0178
GaitSpoofNet-E (Mamba)	0.8247 / 0.1857	0.9500 / 0.0575	**0.9830 / 0.0190**	0.9830 / 0.0190	0.9830 / 0.0190	0.9830 / 0.0190
GaitSpoofNet-F (GRU)	0.8329 / 0.1740	0.9558 / 0.0482	**0.9840 / 0.0165**	0.9840 / 0.0165	0.9840 / 0.0165	0.9840 / 0.0165
GaitSpoofNet-G (LSTM)	0.8268 / 0.1753	0.9493 / 0.0526	**0.9839 / 0.0178**	0.9839 / 0.0178	0.9839 / 0.0178	0.9839 / 0.0178
GaitSpoofNet-H (Insp. Mamba)	0.8283 / 0.1793	0.9504 / 0.0542	**0.9805 / 0.0214**	0.9805 / 0.0214	0.9805 / 0.0214	0.9805 / 0.0214

Bold values indicate the best performance.

**Table 9:** Temporal ablation study: impact of sequence length (T) on accuracy and EER (disjoint split).

Model	T=1 (Acc / EER)	T=10 (Acc / EER)	T=20 (Acc / EER)	T=50 (Acc / EER)	T=100 (Acc / EER)
GaitSpoofNet-E (Mamba)	0.7698 / 0.2468	0.8892 / 0.1267	0.9350 / 0.0756	**0.9589 / 0.0505**	0.9589 / 0.0505
GaitSpoofNet-F (GRU)	0.8004 / 0.2118	0.8901 / 0.1271	**0.9171 / 0.0933**	0.9171 / 0.0933	0.9171 / 0.0933
GaitSpoofNet-G (LSTM)	0.8032 / 0.2083	0.8899 / 0.1196	**0.9173 / 0.0905**	0.9173 / 0.0905	0.9173 / 0.0905
GaitSpoofNet-H (Inspired Mamba)	0.7624 / 0.2579	0.8829 / 0.1284	0.9294 / 0.0811	**0.9546 / 0.0539**	0.9546 / 0.0539

Bold values indicate the best performance.

**Table 10:** Feature separability metrics across random and disjoint evaluation scenarios.

Model	Random split		Disjoint split	
	Fisher discriminant ratio	Silhouette score	Fisher discriminant ratio	Silhouette score
GaitSpoofNet-A	1.7619	0.5828	-	-
GaitSpoofNet-B	1.6381	0.5405	-	-
GaitSpoofNet-C	1.4409	0.5201	-	-
GaitSpoofNet-D	3.7443	0.6802	-	-
GaitSpoofNet-E (Mamba)	1.7974	0.5599	1.1767	0.4936
GaitSpoofNet-F (GRU)	N/A^*^	N/A^*^	1.3912	0.5032
GaitSpoofNet-G (LSTM)	N/A^*^	N/A^*^	1.6350	0.5538
GaitSpoofNet-H (Insp. Mamba)	2.967144	0.6545	1.487782	0.5429

^*^N/A: Evaluation batch contained only a single class.

(-): Not evaluated in this scenario.

### 4.6 Cross-condition generalization

We evaluated the models' robustness by testing against isolated spoofing conditions (Tables 11, 12). All models generalized exceptionally well, but consistently showed lower error rates against CL attacks than BG attacks. This aligns with visual error analysis: coats induce severe, full-body silhouette deformation, whereas bags induce only localized edge deformation, making them slightly harder to isolate.

**Table 11:** Cross-condition generalization metrics (random split: normal vs. isolated attack type).

Model	Evaluation subset	Accuracy	ROC-AUC	EER
GaitSpoofNet-A	Normal vs. BG	0.9310	0.9580	0.1110
	Normal vs. CL	0.9549	0.9903	0.0461
GaitSpoofNet-B	Normal vs. BG	0.9443	0.9775	0.0783
	Normal vs. CL	0.9591	0.9856	0.0549
GaitSpoofNet-C	Normal vs. BG	0.9000	0.9689	0.0944
	Normal vs. CL	0.9383	0.9821	0.0519
GaitSpoofNet-D	Normal vs. BG	0.9824	0.9971	0.0219
	Normal vs. CL	0.9887	0.9991	0.0106
GaitSpoofNet-E (Official Mamba)	Normal vs. BG	0.9824	0.9959	0.0245
	Normal vs. CL	0.9891	0.9973	0.0131
GaitSpoofNet-F (GRU)	Normal vs. BG	0.9828	0.9972	0.0228
	Normal vs. CL	0.9888	0.9993	0.0110
GaitSpoofNet-G (LSTM)	Normal vs. BG	0.9824	0.9971	0.0219
	Normal vs. CL	0.9887	0.9991	0.0106
GaitSpoofNet-H (Insp. Mamba)	Normal vs. BG	0.9799	0.9949	0.0268
	Normal vs. CL	0.9877	0.9968	0.0143

**Table 12:** Cross-condition generalization metrics (disjoint split: normal vs. isolated attack type).

Model	Evaluation subset	Accuracy	ROC-AUC	EER
GaitSpoofNet-E (Mamba SSM)	Normal vs. BG	0.9454	0.9727	0.0786
	Normal vs. CL	0.9569	0.9851	0.0514
GaitSpoofNet-F (GRU)	Normal vs. BG	0.9228	0.9581	0.1031
	Normal vs. CL	0.9308	0.9659	0.0826
GaitSpoofNet-G (LSTM)	Normal vs. BG	0.9239	0.9598	0.1006
	Normal vs. CL	0.9307	0.9684	0.0804
GaitSpoofNet-H (Inspired Mamba)	Normal vs. BG	0.9448	0.9720	0.0843
	Normal vs. CL	0.9558	0.9820	0.0588

### 4.7 Statistical significance analysis

To substantiate the comparative performance differences between our evaluated sequence architectures, we conducted a formal pairwise statistical analysis. We utilized the Paired t-test to compare the 5-fold subject-disjoint cross-validation results across our primary metrics (Accuracy, ROC-AUC, EER, and HTER) for the top-performing models (GaitSpoofNet-E, F, G, and H).

The Paired *t*-test was selected as the standard instrument for cross-validation fold comparisons. A non-parametric alternative, such as the Wilcoxon signed-rank test, was explicitly excluded due to the structural mathematical constraints of our 5-fold (*N* = 5) design. In this configuration, the Wilcoxon test enforces a theoretical minimum two-sided *p*-value of 0.0625 (2/2^5^), permanently preventing it from reaching the standard α = 0.05 significance threshold regardless of effect size. The Paired t-test, conversely, retains adequate resolution at α = 0.05 and its approximate-normality assumption on paired metric differences is reasonable in this setting.

As detailed in Table 13, the LSTM architecture (GaitSpoofNet-G) demonstrated statistically significant improvements over the Official Mamba baseline (GaitSpoofNet-E) in Accuracy (*p* = 0.0181), as well as over our Inspired Mamba (GaitSpoofNet-H) in Accuracy (*p* = 0.0212). Furthermore, the GRU architecture (GaitSpoofNet-F) exhibited statistically significant superiority in ROC-AUC when compared to the Official Mamba (*p* = 0.0029). The Inspired Mamba performed statistically similarly to the Official Mamba across all evaluated metrics (*p*≥0.4930), validating that our specific structural adaptations preserved the baseline state-space representation capabilities without degradation. Comparisons such as GRU vs. LSTM (*p* = 0.1666) and GRU vs. Inspired Mamba (*p* = 0.0801) did not reach statistical significance despite appreciable mean gaps, indicating that those specific margin differences may reflect fold-level variance rather than absolute architectural superiority.

**Table 13:** Pairwise statistical significance (paired *T*-test *p*-values) across 5-Fold LNSOCV.

Model Comparison	Accuracy (*p*-value)	ROC-AUC (*p*-value)	EER (*p*-value)	HTER (*p*-value)
Official Mamba vs. GRU	0.1191	0.0029^*^	0.0501	0.0960
Official Mamba vs. LSTM	0.0181^*^	0.1326	0.3780	0.0771
Official Mamba vs. Insp. Mamba	0.4930	0.7739	0.8698	0.9609
GRU vs. LSTM	0.1666	0.4751	0.7297	0.8634
GRU vs. Insp. Mamba	0.1405	0.1379	0.0935	0.0801
LSTM vs. Insp. Mamba	0.0212^*^	0.0900	0.3821	0.1737

^*^Statistically significant at α = 0.05. Bold values indicate the best performance.

## 5 Discussion of gait anti-spoofing results

This study addresses the critical need for gait spoofing models, given the limited number of publications on this topic, and repurposes the well-known CASIA-B dataset for gait spoofing detection. It also tries, for the first time, to evaluate the models under practical application scenarios. So, we use the current dataset with a different splitting strategy to simulate two practical applications. The first splits the dataset randomly, which may allow some subjects to be repeated partially in training and validation, to simulate a public area that anyone can visit. The second strategy is LNSOCV to ensure that no subject is repeated in the training and validation datasets, simulating unauthorized places like military bases. In addition, we proposed eight gait spoofing detection models, called GaitSpoofNet-A, GaitSpoofNet-B, GaitSpoofNet-C, GaitSpoofNet-D, GaitSpoofNet-E, GaitSpoofNet-F, GaitSpoofNet-G and GaitSpoofNet-H. We divided the results into two scenarios: the first scenario simulates gait spoofing detection in public places, and this is conducted and tested on all models. The second scenario is for unauthorized places, and this is tested on the best four model results from the first scenario.

### 5.1 Scenario 1: public places

In this scenario, random splitting was applied to the training and validation datasets, allowing subjects to appear partially in both sets simultaneously. Under these conditions, the final four temporal models achieved superior performance. This scenario simulates gait spoofing detection in public environments. GaitSpoofNet-E, F, G, and H models showed better generalization, meaning they can learn important features that help detect spoofing more reliably in changing environments.

Under the random-split protocol, the same subject can appear in both the training and validation sets, albeit under different condition labels. Consequently, the primary classification challenge is to differentiate between conditions, such as NM and BG/CL, rather than generalizing across unseen subjects. In this scenario, the GRU's lower parameter count helps mitigate overfitting caused by the limited diversity in each subject's appearance. This architectural simplicity provides a distinct advantage over the more complex LSTM cell state, which possesses a higher capacity that can overfit to the limited within-subject variation present in the random-split data.

Since the network learns the spatial appearance distributions of the subjects during training, the primary challenge shifts from subject generalization to condition classification (NM vs. BG/CL). In this context, the simpler GRU gating mechanism provides a distinct advantage:

- **Fewer parameters:** The GRU has 2 gates compared to the LSTM's 3 gates. With the limited within-subject diversity of a 20-frame silhouette sequence, additional capacity (such as the LSTM cell state) can lead to mild overfitting—a phenomenon confirmed by a slightly larger train-validation accuracy gap in GaitSpoofNet-G compared to GaitSpoofNet-F.
- **Faster convergence:** The GRU training loss reaches a plateau approximately 5 epochs earlier than the LSTM, and the CosineAnnealingLR scheduler exploits this faster convergence to deliver a slightly better final learning-rate schedule.
- **Sufficient temporal span:** A gait cycle in CASIA-B spans approximately 15–20 frames at a standard capture rate. The GRU update gate *z*_*t*_ is sufficient to propagate cycle-level context across 20 frames without the longer-range memory buffer that the LSTM's cell state provides.
- **Attention complements GRU:** The soft-attention mechanism compensates for the GRU's weaker long-range memory by selectively pooling the most discriminative frames. The GRU produces more varied attention weights (higher entropy) than the LSTM in Scenario 1, implying it attends more selectively to the peak-spoofing-evidence frames (e.g., full-coat silhouette frames where BG/CL diverges most from NM).

### 5.2 Scenario 2: unauthorized places

LNSOCV was used to ensure no subject overlap between the training and validation datasets. In this scenario, we selected the best four models from the first scenario and re-evaluated them in a more challenging setup that simulates restricted-access areas, accessible only to authorized personnel. Based on the results, GaitSpoofNet-G is the best model among the four. It performed better than GaitSpoofNet-E and GaitSpoofNet-F in most evaluation metrics. It had the highest accuracy (0.8986), the lowest HTER (0.1072) and BPCER (0.0803), and the highest TPR@1%FPR (0.5287), making GaitSpoofNet-G the strongest overall model.

To rigorously validate the statistical significance of these results across the 5 subject-disjoint folds, we calculated the overall 95% Confidence Interval (CI) for the mean accuracy using the Student's T-distribution. The baseline LSTM (GaitSpoofNet-G) achieved the highest mean accuracy at 89.86% (95% CI: 87.40%–92.32%). Our pairwise statistical analysis confirms its superior generalization to unseen subjects when compared to the Official Mamba (*p* = 0.0181) and Inspired Mamba (*p* = 0.0212) architectures. While the LSTM also exhibited a higher mean accuracy than the GRU (89.60%), the difference between the two recurrent models did not reach statistical significance (*p* = 0.1666), indicating that both recurrent architectures offer highly robust and statistically comparable subject-disjoint generalization.

The decline in accuracy from approximately 0.98 in Scenario 1 to roughly 0.90 in Scenario 2 is attributed to the removal of subject overlap between the training and validation sets. In the random-split protocol, each subject's silhouette distribution is partially observed during training. This enables the model to leverage subject-specific features–such as body proportions, silhouette width, and stride geometry–as auxiliary cues for classification alongside condition-related temporal features, resulting in identity leakage. In contrast, the LNSOCV protocol ensures that validation subjects remain completely unseen during training, effectively eliminating this identity-based bias. Consequently, the model must rely solely on subject-independent temporal spoofing cues.

In the subject-disjoint LNSOCV protocol, validation subjects are entirely unseen during training. The model must generalize across individuals with different body proportions, natural stride lengths, and silhouette qualities. Here, the LSTM's explicit cell state provides a decisive advantage:

- **Long-range temporal memory:** The cell state *c*_*t*_ can maintain subject-independent temporal patterns (e.g., gait cycle periodicity, within-cycle posture trajectory) across the full 20-frame sequence. When the feature vector *f*_*t*_ from a novel subject contains appearance statistics not seen in training, the cell state provides inertia that prevents the catastrophic forgetting of the spoofing-discriminative temporal pattern.
- **Forget gate as a subject-invariant filter:** The forget gate *f*_*t*_ learns to suppress subject-specific appearance variance (such as body size or silhouette edge noise) while retaining the condition-discriminative temporal signal (e.g., the characteristic silhouette bulge of a coat versus a normal contour). The GRU's reset gate *r*_*t*_ performs a similar function but without a separately maintained cell state, leading to a less stable representation under high subject variation.
- **Training convergence under cross-subject generalization:** The LSTM training loss in Scenario 2 converges more slowly but achieves a lower final EER, consistent with a model that is building a richer internal representation. The cell state gradient path in the LSTM is longer and less prone to vanishing than the GRU equivalent, enabling better gradient flow across the 20-frame sequence.

While our work provides a solid foundation for detecting appearance-based spoofing, it is important to recognize the broader landscape of gait security. The attacks we simulated (wearing coats or carrying bags) are purely physical. They do not cover digital presentation attacks, like deepfakes generated by GANs (Jia et al., 2019) or adversarial attacks specifically crafted to trick neural networks (Zhu et al., 2021; Guo et al., 2020). Additionally, scenarios where an attacker actively tries to mimic someone else's walk (Hadid et al., 2012; Bustard et al., 2014; Hadid et al., 2015) introduce complex dynamic shifts that are not fully captured by standard covariate datasets. Furthermore, looking at real-world forensic applications, gait analysis often has to deal with heavy occlusion and drastic changes in camera angles. To tackle these specific spatial issues, the field has seen great success with advanced techniques like 3D human body reconstruction (Meng et al., 2023), 3D view deformation (Ammar Alsherfawi Aljazaerly et al., 2025), and neural networks specifically designed to handle occlusion (Kumar et al., 2024).

In light of these advancements, our study aims to fulfill a specific, practical role: serving as an efficient, real-time 'first line of defense' for standard 2D surveillance systems. While complex 3D models are robust and remain the gold standard for offline forensic investigations, our 2D temporal models—specifically Mamba, GRU, and LSTM—are significantly more efficient. Consequently, they can flag anomalies instantaneously across existing camera networks without the high computational cost of 3D rendering. Moving forward, a promising direction would be to integrate our lightweight temporal anomaly detection with 3D skeleton and mesh frameworks. Such a hybrid approach could lead to a comprehensive security system capable of withstanding both physical disguises and advanced digital deepfakes.

While this study focuses on vision-based PAD, it is important to compare these results to those of sensor-based gait authentication. Wearable and smartphone sensors (using accelerometers and gyroscopes) capture 3D microdynamics and physical forces that are naturally resistant to visual concealment, such as clothing modifications. Sensor data offers a significant advantage in detecting appearance-based spoofing because it relies on the wearer's actual biomechanics rather than their external silhouette. However, sensor-based systems require subject cooperation as well as device deployment. In contrast, our vision-based models operate non-intrusively from a distance, relying solely on spatiotemporal modeling to distinguish genuine presentations from malicious attacks. The superior ROC-AUC scores achieved by our GRU in the open-access scenario (0.9983) and our LSTM in the restricted-access scenario (0.9477) demonstrate that deep temporal analysis can effectively bridge the existing robustness gap. This enables vision-based systems to achieve high levels of security without the need for wearable hardware.

While CASIA-B provides a solid foundation for studying appearance-based spoofing, it does not fully address the unpredictable environments found in practical security settings. Authentic surveillance footage often features changing lighting, cluttered backgrounds, and varied camera angles. Since we tested our temporal models in a more controlled environment, we still do not know how they will perform in these complex everyday situations. To truly gauge the practical reliability of these PAD systems, researchers will need to test them against a much wider range of environmental factors. They could use existing recognition datasets, such as OU-ISIR and FVG, to assess how well the models handle changes in lighting and viewpoints. Ultimately, the field needs a dedicated, publicly available gait PAD benchmark that includes various real-world environments with subjects who actively try to deceive the system. For use in highly secure areas, these vision-based temporal models should not operate independently. Combining gait anomaly detection with other biometrics, such as face or voice recognition, would create a much stronger and more reliable defense system.

Finally, for practical and highly secure deployment in operational settings, these vision-based gait anti-spoofing mechanisms should ultimately be integrated with multimodal biometrics—such as combining gait anomaly detection with face or voice recognition–to establish a comprehensive, multi-layered defense architecture.

## 6 Conclusion on gait anti-spoofing advancements

This work addresses the critical challenge of spoofing in gait-based authentication systems by investigating advanced spatio-temporal models for Presentation Attack Detection (PAD). In this paper, gait spoofing is defined as the deliberate alteration of an individual's appearance through clothing or carrying conditions with the intent to impersonate or deceive the gait authentication system, a type of presentation attack. This study further distinguishes itself by evaluating spoofing detection models in practical scenarios, including a public-access scenario with random dataset splitting and a restricted-access scenario employing LNSOCV strategy, an experimental design often neglected in previous work. This paper addressed the critical challenge of developing robust anti-spoofing mechanisms for gait-based biometric systems. We conducted a comprehensive comparative investigation into the effectiveness of advanced spatio-temporal architectures—specifically the official Mamba Selective State Space Model, GRU, and LSTM models—for their ability to detect presentation attacks. By repurposing the CASIA-B dataset, this work establishes the foundational benchmark against which future silhouette-based PAD models can be measured. Our experimental results conclusively demonstrated that these advanced temporal models significantly enhance gait spoofing detection capabilities. Among the evaluated architectures, the GRU-based model emerged as the most effective for anti-spoofing in open-access environments, achieving a state-of-the-art final validation accuracy of 0.9840 and an ROC-AUC of 0.9983. On the other hand, in restricted-access scenarios, the LSTM-based model achieved the highest mean validation accuracy of 0.8986 and an ROC-AUC of 0.9477, performing statistically similarly to the GRU while demonstrating statistically significant improvements over the baseline State-Space representation. These findings highlight the substantial potential of advanced recurrent and state-space models to fortify gait recognition systems against the pervasive threat of spoofing. The superior anti-spoofing performance of the GRU architecture in open-access environments, and the effectiveness of the LSTM model in restricted-access scenarios, provide a strong foundation for building more secure and adaptable gait-based authentication solutions. This comparative analysis offers critical insights for the ongoing advancement of biometric security against sophisticated presentation attacks. Despite these advancements, the field remains limited by a reliance on repurposed datasets. Therefore, future work must prioritize the creation of a dedicated, public gait PAD benchmark featuring subjects with verified adversarial intent. Such a dataset is essential to accurately evaluate and advance the real-world robustness of gait anti-spoofing mechanisms.

## Data Availability

The original contributions presented in the study are included in the article/Supplementary material, further inquiries can be directed to the corresponding author.
