# Supplementary material for: GaitSpoofNet: advanced spatio-temporal architectures for vision-based presentation attack detection
Source: Front Artif Intell. 2026 Jun 30;9:1821341. doi: 10.3389/frai.2026.1821341 (PMC13365055; doi:10.3389/frai.2026.1821341)
Supplement: Supplementary file 1 [file Data_Sheet_1.pdf]

1 TEMPORAL SEQUENCE MODELS

When performing a comparative analysis of advanced spatio-temporal architectures—especially for resilient sequential modeling in PAD it is essential to specify how each model encapsulates temporal dynamics and regulates its internal state over time. This section discusses the theoretical foundations of the Long Short-Term Memory (LSTM), Gated Recurrent Unit (GRU), and the Mamba Selective State Space Model (SSM). The structural differences between these units are illustrated in Figure 1, while their operational characteristics are contrasted in Table 1.

1.1 Long Short-Term Memory (LSTM)

Long Short-Term Memory (LSTM) networks, initially proposed by Hochreiter and Schmidhuber , are a specialized deep learning architecture designed to overcome the vanishing gradient problem inherent in standard Recurrent Neural Networks (RNNs). They achieve this by introducing a dual-state mechanism consisting of a hidden state (h_t) and a cell state (C_t). The cell state acts as an internal memory conveyor belt, allowing gradients to flow relatively uninterrupted across long temporal sequences, making them ideal for tasks involving complex sequential data.

Given an input sequence X_t at time step t and the previous hidden state h_{t-1}, the LSTM strictly regulates the flow of information through three specialized gates (forget, input, and output) via the following operations:

$$f_t = \sigma(W_f \cdot [h_{t-1}, X_t] + b_f) \tag{1}$$

Forget Gate (f_t): Determines what proportion of the previous memory to discard based on the current input and past hidden state.

$$i_t = \sigma(W_i \cdot [h_{t-1}, X_t] + b_i) \tag{2}$$

Input Gate (i_t): Decides which specific values within the memory state will be updated with new information.

$$\tilde{C}_t = \tanh(W_C \cdot [h_{t-1}, X_t] + b_C) \tag{3}$$

Candidate State ($\tilde{C}_t$): Generates a vector of new, normalized candidate values that could potentially be added to the cell state.

$$C_t = f_t \odot C_{t-1} + i_t \odot \tilde{C}_t \tag{4}$$

Cell State Update (C_t): Computes the new internal memory by dropping the chosen old data and adding the scaled candidate data.

$$o_t = \sigma(W_o \cdot [h_{t-1}, X_t] + b_o) \tag{5}$$

Output Gate (o_t): Determines which specific parts of the updated cell memory will be exposed to the next network layer.

$$h_t = o_t \odot \tanh(C_t) \tag{6}$$

Hidden State Update (h_t): Calculates the final hidden representation for the current time step by filtering the cell state through the output gate.

$$y_t = h_t \tag{7}$$

Final Output (y_t): For structural consistency across sequence models, the final observable output at this step is taken directly as $y_t = h_t$. (Note: σ denotes the sigmoid activation, $\odot$ is element-wise multiplication, and W and b are learnable weights and biases).

1.2 Gated Recurrent Unit (GRU)

Gated Recurrent Units (GRUs) are a streamlined variant of the LSTM architecture that combines the standard input and forget gates into a single update gate, reducing parameter overhead and computational load. This update gate determines how much previous information will be retained

for future steps, while a complementary reset gate controls how much past data will be discarded in order to prioritize current relevance. By simplifying these gating mechanisms, GRUs achieve an optimal balance of efficiency and performance. They frequently match or exceed LSTM accuracy while demanding fewer computational resources and training much faster. Nevertheless, they remain constrained by sequential dependencies that restrict parallelization.

The state updates for a GRU at time step t are defined as:

$$r_t = \sigma(W_r \cdot [h_{t-1}, X_t] + b_r) \tag{8}$$

Reset Gate (r_t): Determines how much of the past information to forget, allowing the model to drop irrelevant history.

$$z_t = \sigma(W_z \cdot [h_{t-1}, X_t] + b_z) \tag{9}$$

Update Gate (z_t): Controls the interpolation between the old state and the new candidate state, combining the roles of the LSTM's forget and input gates.

$$\tilde{h}_t = \tanh(W \cdot [r_t \odot h_{t-1}, X_t] + b) \tag{10}$$

Candidate State ($\tilde{h}_t$): Proposes a new hidden state based on the current input and the explicitly retained past state.

$$h_t = (1 - z_t) \odot h_{t-1} + z_t \odot \tilde{h}_t \tag{11}$$

Final Hidden State (h_t): Blends the old and new states to compute the final hidden representation for the current time step.

$$y_t = h_t \tag{12}$$

Final Output (y_t): For consistency, the observable output representation is taken as $y_t = h_t$.

Table 1 summarizes the fundamental structural and operational differences between the evaluated models.

1.3 Mamba (Selective State Space Model)

Mamba is a highly efficient neural network architecture derived from State Space Models (SSMs). Demonstrating high inference speeds and low memory requirements for long sequences, it has emerged as one of the first competitive alternatives to transformer models for sequence modeling tasks.

Unlike traditional RNNs, Mamba introduces a *Selective* mechanism, allowing the model to filter information dynamically based on the input. By making the continuous-to-discrete parameters functions of the input X_t, Mamba selectively remembers or ignores information at each token without utilizing self-attention matrices.

A continuous State Space Model operates on a latent state $h(t)$:

$$h'(t) = Ah(t) + Bx(t) \tag{13}$$

$$y(t) = Ch(t) \tag{14}$$

To apply this to discrete sequences, Mamba discretizes the continuous parameters. Crucially, intermediate parameters B_t, C_t, and the step size Δ_t are obtained via linear projections of the input X_t. As depicted structurally (note that the discretization step is abstracted in the diagram for visual clarity), the projected step size Δ_t drives the continuous-to-discrete transformation, effectively parameterizing the time-varying discrete matrices $\bar{A}$ and $\bar{B}$:

$$\bar{A} = \exp(\Delta_t A) \tag{15}$$

Discrete Transition Matrix ($\bar{A}$): Determines how the hidden state evolves based on the input-dependent step size.

$$\bar{B} = (\Delta_t A)^{-1}(\exp(\Delta_t A) - I) \cdot \Delta_t B_t \tag{16}$$

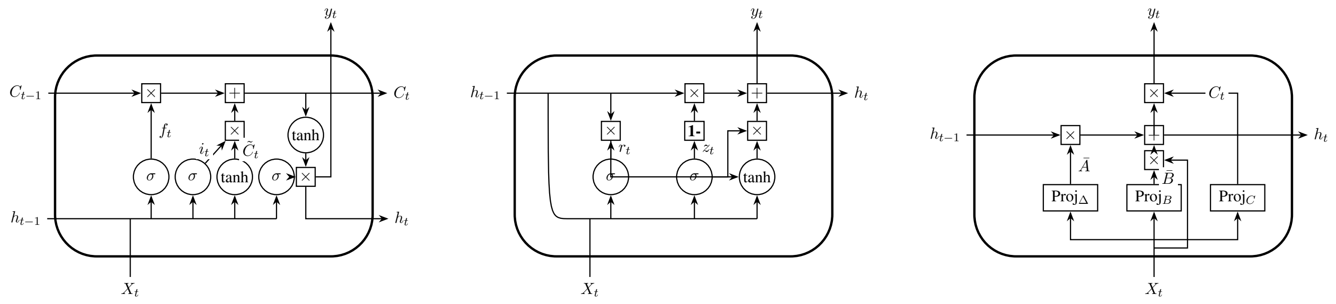

Figure 1. General structure of LSTM (left), GRU (middle), and Mamba (right) units.

Table 1. Summary Comparison of LSTM, GRU, and Mamba Architectures

Feature	LSTM	GRU	Mamba (Selective SSM)
**State Tracking**	Dual states (C_t, h_t)	Single state (h_t)	Single continuous/ discrete state (h_t)
**Control Mechanism**	3 Gates (Input, Output, Forget)	2 Gates (Update, Reset)	Selective input-dependent parameters (Δ, B, C)
**Complexity**	High (More parameters)	Medium (Fewer parameters)	High (Requires hardware-aware algorithms)
**Parallelization**	Sequential (Not parallelizable)	Sequential (Not parallelizable)	Highly parallelizable via parallel scan
**Long-Sequence Capability**	Good, but degrades eventually	Good, similar to LSTM	Excellent, specifically optimized for long contexts

Discrete Input Matrix ($\bar{B}$): Filters the continuous input into the discrete state space (assuming A is invertible).

$$h_t = \bar{A}h_{t-1} + \bar{B}X_t \tag{17}$$

Hidden State Update (h_t): Incorporates the selectively filtered input into the temporal sequence.

$$y_t = C_t h_t \tag{18}$$

Output Projection (y_t): Maps the selectively updated hidden state to the final observable output.
